# The Role of Attention Shifting in Orthographic Competencies: Cross-Sectional Findings from 1st, 3rd, and 8th Grade Students

**DOI:** 10.3389/fpsyg.2017.01665

**Published:** 2017-09-26

**Authors:** Antje von Suchodoletz, Anika Fäsche, Irene T. Skuballa

**Affiliations:** ^1^Department of Psychology, University of Freiburg, Freiburg, Germany; ^2^Department of Psychology, New York University Abu Dhabi, Abu Dhabi, United Arab Emirates

**Keywords:** attention shifting, spelling, cross-sectional study, elementary school children, secondary school children, gender differences, cohort study

## Abstract

Attention shifting refers to one core component of executive functions, a set of higher-order cognitive processes that predict different aspects of academic achievement. To date, few studies have investigated the role of attention shifting in orthographic competencies during middle childhood and early adolescence. In the present study, 69 first-grade, 121 third-grade, and 85 eighth-grade students' attention shifting was tested with a computer version of the Dimensional Change Card Sort (DCCS; Zelazo, 2006). General spelling skills and specific writing and spelling strategies were assessed with the Hamburger Writing Test (May, 2002). Results suggested associations between attention shifting and various orthographic competencies that differ across age groups and by sex. Across all age groups, better attention shifting was associated with less errors in applying alphabetical strategies. In third graders, better attention shifting was furthermore related to better general spelling skills and less errors in using orthographical strategies. In this age group, associations did not differ by sex. Among first graders, attention shifting was negatively related to general spelling skills, but only for boys. In contrast, attention shifting was positively related to general spelling skills in eighth graders, but only for girls. Finally, better attention shifting was associated with less case-related errors in eighth graders, independent of students' sex. In sum, the data provide insight into both variability and consistency in the pattern of relations between attention shifting and various orthographic competencies among elementary and middle school students.

## Introduction

Attention shifting, one core component of executive functions, is defined as the ability to flexibly shift “back and forth between multiple tasks, operations, or mental sets” (Miyake et al., 2000, p. 55). Spelling mastery appears to require children to flexibly shift between multiple demands that are embedded in the process of transforming a spoken word into written symbols (Lubin et al., 2016). For example, recognizing smaller units of meaning and sound, retrieving the correct letter or letter combination for each sound, and finally writing the letter in the correct form requires the flexible shifting of attention (Aram et al., 2014; Blair and Raver, 2015). In addition, spelling requires one to shift between strategies, lexical and non-lexical strategies in particular, when decoding and spelling words (Sheriston et al., 2016).

The ability to voluntarily focus or shift attention as needed develops during the early elementary school years, between 7 and 9 years of age (Anderson, 2010). Attention shifting continues to improve throughout middle childhood and becomes relatively mature by the beginning of adolescence (Anderson, 2010). A variety of measures exist to assess attention shifting. However, only few can be used at different stages of the lifespan and across age groups, one of which is the computer-based version of the Dimensional Change Card Sort (DCCS; Zelazo, 2006). The DCCS requires participants to sort objects by two dimensions, color and shape. Preschool children are able to switch tasks as long as the stimuli vary along only one dimension (Diamond et al., 2005). As they grow older, accuracy on the DCCS increases, as children are able to switch from sorting by either color or shape to sorting by the other (Diamond et al., 2005). Most children are able to complete the DCCS accurately around the age of school entry (Diamond and Kirkham, 2005). However, there is evidence that, despite high accuracy rates in sorting objects by switching dimensions, attentional inertia persists. Diamond and Kirkham (2005) used a computer-based version of the DCCS with young adults (i.e., undergraduate college students in their early twenties). While the participants were able to switch sorting dimensions, their reaction time pattern was similar to the accuracy pattern among young children, i.e., reaction time was significantly slower when the sorting criterion changed (Diamond and Kirkham, 2005). Evidence that the computer-based version of the DCCS captures individual differences in attention shifting among children and adults suggests that the DCCS is an appropriate measure for investigating attention shifting at different stages of the life span and across a wide age range.

### The acquisition of spelling

Spelling is an important prerequisite for competent writing and predicts a number of literacy outcomes at later ages (Temple et al., 1982; Aram et al., 2014). Spelling in alphabetic orthographies can be defined as the ability to transform a spoken word into written symbols on the page (Berninger et al., 1996). Learning to spell means being able to map phonemes (i.e., units of speech) onto letters (i.e., units of print), and to understand that letters primarily represent sounds in language rather than meaning (McBride-Chang, 2004; Aram et al., 2014). Three sequential schemes of early spelling development have been suggested: graphic, writing-like, and symbolic writing (Levin and Bus, 2003). Writing in the graphic phase is characterized by the spontaneous production of small graphic forms and shapes. As soon as children know that letters, not pictures or shapes, represent print units, they move to the next phase, although they might not yet understand the relation between letter names and their sounds (McBride-Chang, 2004). During the preschool period, children discover writing-like features (Temple et al., 1982). Once they reach the phase of symbolic writing children are able to use symbolic units and move from phonetic writing to conventional spelling (Temple et al., 1982; Levin and Bus, 2003).

Although, in alphabetic orthographies letters typically map onto phonemes, the writing system also contains non-alphabetic aspects (Nagy et al., 2006). As children learn to spell, they acquire knowledge about morphology and orthographic patterns. Such knowledge is successively incorporated in children's attempts to spell as they learn to conform to the standard spelling rules of their language (McBride-Chang, 2004). Most research on spelling acquisition has focused on the early childhood years. However, spelling development continues after school entry across all years of schooling when children are increasingly confronted with words of irregular spelling patterns, abstract words, and complex clause types that require specialized knowledge of spelling rules (Temple et al., 1982; McBride-Chang, 2004; Christie and Derewianka, 2010). While children have some basic morphological knowledge as early as in first grade (Treiman and Cassar, 1996), their use of morphological strategies is still fragile and not reliably reflected in their spellings until after third grade (Nagy et al., 2006). Similarly, basic orthographic knowledge emerges early in spelling acquisition, i.e., in kindergarten and first grade (Treiman and Bourassa, 2000). It is not until the later school years, however, that children can reliably incorporate their knowledge about orthographical strategies in their spellings (Treiman and Bourassa, 2000). For example, knowledge of allowable consonant doublets (i.e., two-letter spellings that typically occur in the middle and at the end of words) emerges in first grade, but proficiency in applying knowledge of orthographic patterns is not reached until sixth grade and above (Cassar and Treiman, 1997; Treiman and Bourassa, 2000). However, spelling is typically studied from a word-level perspective, thus limiting conclusions about the role of morphology and knowledge of orthographic units in spelling.

### Attention shifting and spelling

Early attention shifting supports young children's acquisition of precursor skills to the development of later spelling, such as letter/alphabet knowledge and print awareness (Blair and Razza, 2007; Bierman et al., 2008). As children grow older and enter formal schooling, attention shifting helps them to develop adaptive learning strategies and apply them flexibly to changing task demands. During the early elementary school years, children are increasingly confronted with non-alphabetic aspects of the writing system that require them to flexibly shift between several mental tasks, including retrieving the spelling of words from memory, applying orthographic patterns, or using phoneme-grapheme correspondence rules (Lubin et al., 2016). Although, various executive function components contribute to spelling acquisition, Lubin et al. (2016) found that attention shifting seems to be particularly predictive of spelling outcomes among elementary school children. Fourth-grade children were administered the Creature Counting subtest from the Test of Everyday Attention for Children (Manly et al., 1999) to measure attention shifting. The test requires children to use arrows as a cue to switch the direction of their counting. Spelling outcomes were assessed with a dictation test. After controlling for child age, sex, and nonverbal intelligence, executive function skills explained 19% of the variance in the spelling outcome. However, the findings indicated that only attention shifting significantly contributed to explaining the variance in children's spelling skills.

While the relation between attention shifting and literacy skills is well-established in samples of English-speaking children (Blair and Razza, 2007; Bierman et al., 2008), there is some evidence suggesting that associations might be different in other languages. Among French-speaking kindergarten children, for example, attention shifting and emergent literacy skills were not significantly associated (Monette et al., 2011). In another study with a sample of Dutch-speaking elementary school students, the relation between executive functions and reading was found to be negative (van der Sluis et al., 2007).

### Sex differences in spelling and attention shifting

The sex achievement gap suggests that boys may be at greater risk for school difficulties than girls (Matthews et al., 2009; Rimm-Kaufman et al., 2009; DiPrete and Jennings, 2012; Wanless et al., 2013). Girls frequently outperform boys on a wide range of measures of school achievement across different learning domains. For reading, for example, girls' advantage corresponds to approximately one school year's progress [PISA^1^ 2009 study: Organization for Economic Co-operation and Development (OECD), 2010]. Girls also demonstrated higher writing competence (Pajares and Valiente, 1999) and written orthographic fluency (Berninger and Fuller, 1992) compared to boys. The pattern of sex differences in spelling skills among typically developing writers was also replicated in samples of children with dyslexia and their parent with dyslexia (Berninger et al., 2008a). In both samples, the male participants were consistently more impaired than their female counterparts in measures of spelling and orthographic skills. In search of an explanation, McGeown et al. (2013) argue that sex differences may be related to differences in girls' and boys' strategy preferences and their ability to use strategies effectively. Strategies may be specific to the learning domain (such as orthographical spelling strategies) or domain-general (such as executive functions). Individual differences in executive functions might be related to sex differences in orthographic competencies (Berninger et al., 2008b). Indeed, there is considerable research to suggest that girls may be more efficient at using executive functions (Wilson, 2003; Sabbagh et al., 2006; Matthews et al., 2009; Rimm-Kaufman et al., 2009; Wanless et al., 2013). Specifically, sex differences in attention shifting in favor of girls have been reported across age groups (Klenberg et al., 2001) and various measures of attention shifting (Klenberg et al., 2001; Wilson, 2003). Neuropsychological differences may underlie girls' advantage in attention shifting. Feng et al. (2011) found greater brain activation (interpreted as the use of more attention resources) for women compared to men when completing an attention shifting task among students in their early twenties (mean age in both groups was 21.9 years).

Together, these studies suggest that both spelling and attention shifting skills differ by sex. However, it remains unclear whether there are (a) sex differences in the relation between attention shifting and spelling, and whether (b) sex differences are similar or different across age groups. While some research suggests that the gap between boys and girls in executive functions increases from childhood to adolescence (Else-Quest et al., 2006; Matthews et al., 2009), other evidence indicates that boys could catch up in their executive function skills as they grow older (Gunzenhauser and von Suchodoletz, 2015).

### The present study

The goal of the present study was to investigate whether attention shifting, one core component of executive functions, is equally important for orthographic competencies at different ages and for boys and girls. By using the same measure of attention shifting (a computer version of the DCCS) and grade-appropriate versions of the same spelling task in a sample of first, third, and eighth graders, the study aimed to provide new information on individual differences in attention shifting in middle childhood and early adolescence and its associations with spelling competencies. By doing so, we addressed gaps of prior research regarding differences in task characteristics that limited strong conclusions (Best et al., 2011; Cuevas et al., 2014). The selection of age groups was based on previous literature suggesting that voluntary attention shifting starts to emerge during the early elementary school years and becomes relatively mature by the beginning of adolescence (Anderson, 2010). Moreover, the contribution of attention shifting might depend on the specific outcome being measured. We therefore investigated general spelling skills as well as specific spelling skills, including alphabetical, orthographical, and morphological strategies. Understanding these associations can help to determine the extent to which attention shifting relates to which particular aspect of spelling.

Two research questions guided the present study: (1) Does attention shifting relate to orthographic competencies across all age groups? Specifically, we tested for cross-group invariance between first, third, and eighth graders. (2) Are there sex differences in the relation between attention shifting and orthographic competencies within each age group? We tested for cross-group invariance regarding sex within the samples of first, third, and eighth graders.

## Methods

### Participants and procedure

The participants were 275 school-aged children (51% girls) from South-West Germany. Sixty-nine students were in Grade 1, 121 in Grade 3, and 85 in Grade 8. The students' mean age was 7.23 years in Grade 1 (*SD* = 0.39), 8.47 years in Grade 3 (*SD* = 0.45), and 13.99 years in Grade 8 (*SD* = 0.40). All of the children were typically developing insofar that they were not enrolled in special education or special needs programs at their school. The first- and third-grade students were recruited from public primary schools; the eighth-grade students were recruited from the highest track of the public German secondary school system, i.e., the Gymnasium. For 78% of the first graders, 68% of the third graders, and 87% of the eighth graders, German was the primary language spoken at home.

To ensure that the protocol conforms to ethical standards, the study protocols in Grade 1 and Grade 3 were reviewed by the ethic committee of the German Psychological Association (DGPs). The study protocol in Grade 8 was reviewed by the Department of Psychology, Educational, and Developmental Psychology, at the University of Freiburg, Germany. All of the participants were recruited in their schools. Data was collected only from the children whose parents gave their informed written consent. Recruitment differences resulted in different sizes of the subsamples, in particular, the larger sample of the third-grade students. The third-grade students represent a randomly selected subsample drawn from a sample of over 700 students recruited from 56 classrooms in 34 schools (details can be found elsewhere: von Suchodoletz et al., 2015) who completed an additional data collection that included the measures used for the present analyses. The participants in Grade 1 were recruited from a database of families who participated in research when the children were younger (details can be found elsewhere: Gestsdottir et al., 2014) and had volunteered to be contacted for future research. The participants in Grade 8 were recruited specifically for this study.

The first-grade and third-grade students were tested at the local university, whereas the eighth-grade students were tested at their school. The children were administered the writing test first, followed by the computer version of the DCCS (presented on a laptop). It took between 30 and 45 min to complete the tasks. All of the participants received small incentives for their participation in the study.

### Measures

#### Attention shifting

A computer-based version of the DCCS was used to measure attention shifting (DCCS; Diamond and Kirkham, 2005; Zelazo, 2006; Blankson and Blair, 2016). The DCCS has been shown to be a reliable and valid measure of attention shifting across a wide range of ages. For example, children's performance on the DCCS correlates with other measures of executive functions (Zelazo, 2006). The computer-based version of the DCCS has also been used with adult populations and has been proven to reliably capture individual differences in attention shifting (accuracy and reaction time) among adults (Diamond and Kirkham, 2005; von Suchodoletz et al., 2017).

Stimuli were presented on a laptop screen. The task required the participants to match a target stimulus presented at the top of the screen with two pictures that varied along two dimensions, i.e., color and shape, and appeared at the bottom corners of the screen. To match the pictures, the participants were instructed to press one of two yellow-marked keys on opposite sides of the laptop keyboard to indicate the location of their selection. In addition, a word (either color or shape) was presented at the top of the screen and spoken by a prerecorded voice to cue the participants to match the target picture with the correct corresponding picture on the bottom of the screen. Following a practice trial block, the participants were first asked to correctly sort the stimuli by one dimension (e.g., sort by shape; pre-switch block) and then to switch and sort the stimuli by the other dimension (e.g., color; post-switch block). The final block consisted of mixed trials.

The participants' accuracy (i.e., percent correct) and reaction times (averaged for all correct trials) were recorded across the pre-switch, post-switch, and mixed-block. Trials in which the response was registered earlier than 200 ms or later than 3,000 ms after the onset of the stimulus were excluded from the analyses (Diamond and Kirkham, 2005). The mean percentage of correct responses, ranging between 85 and 95% across the participants (see Table 1), was similar to a study with undergraduate college students (Diamond and Kirkham, 2005). In the current study, attention shifting was measured in terms of inverse efficiency, i.e., average reaction time for all correct trials divided by accuracy (Spence et al., 2001; Schicke et al., 2009). Inverse efficiency scores provide a more psychometrically accurate representation of processing efficiency than using accuracy (i.e., proportion of correct responses) and reaction time as separate variables (Yang et al., 2014). The assumption underlying inverse efficiency scores is that “differences in reaction time performance would decrease if differences in accuracy [were] large but would remain the same if accuracy [were] identical” (Ding et al., 2014, p. 91). Inverse efficiency scores account for possible speed-accuracy trade-offs (i.e., slow responses, but less errors, and vice versa; Spence et al., 2001; Kitagawa and Spence, 2005; Holmes et al., 2007; Schicke et al., 2009). In the present data, a lower inverse efficiency score reflected better attention shifting skills.

**Table 1:** Descriptive statistics for variables of interest.

	**Grade 1**			**Grade 3**			**Grade 8**		
	**All (*n* = 69) *M (SD)***	**Girls (29) *M (SD)***	**Boys (*n* = 40) *M (SD)***	**All (*n* = 121) *M (SD)***	**Girls (*n* = 59) *M (SD)***	**Boys (*n* = 62) *M (SD)***	**All (*n* = 84) *M (SD)***	**Girls (*n* = 51) *M (SD)***	**Boys (*n* = 33) *M (SD)***
**ATTENTION SHIFTING**									
Reaction time correct trials	1520.26	1637.40	1449.97	962.88	1003.49	923.06	686.18	676.27	701.51
	(346.98)	(359.27)	(326.53)	(154.25)	(145.12)	(153.88)	(133.18)	(136.67)	(128.14)
Percent correct	0.92	0.95	0.90	0.86	0.87	0.85	0.92	0.91	0.94
	(0.10)	(0.07)	(0.11)	(0.07)	(0.06)	(0.07)	(0.05)	(0.05)	(0.04)
Inverse efficiency score	1678.44	1752.20	1634.19	1127.35	1156.96	1098.31	747.57	746.86	748.66
	(447.51)	(485.13)	(427.44)	(215.82)	(192.83)	(234.48)	(146.33)	(152.22)	(139.03)
**ORTHOGRAPHIC COMPETENCIES**									
Correct words	7.09	6.96	7.18	23.81	23.36	24.26	46.02	45.55	46.76
	(2.68)	(2.83)	(2.60)	(6.88)	(6.71)	(7.07)	(2.55)	(2.67)	(2.19)
Correct graphemes	48.28	49.00	47.77	175.50	174.28	176.72	335.79	335.02	336.97
	(9.18)	(7.04)	(10.47)	(10.63)	(10.23)	(10.96)	(4.29)	(5.14)	(2.04)
Alphabetical errors	1.51	1.61	1.45	1.47	1.29	1.66	0.56	0.63	0.45
	(2.05)	(2.17)	(1.99)	(1.65)	(1.50)	(1.78)	(0.61)	(0.56)	(0.67)
Orthographical errors	5.76	5.96	5.63	4.23	4.53	3.93	1.08	1.14	1.00
	(2.09)	(2.12)	(2.08)	(3.69)	(3.68)	(3.72)	(1.29)	(1.39)	(1.15)
Morphological errors	–	–	–	–	–	–	0.57	0.57	0.58
							(0.80)	(0.78)	(0.83)
Redundant elements	0.63	0.54	0.70	2.20	2.09	2.31	–	–	–
	(0.62)	(0.58)	(0.65)	(1.99)	(1.76)	(2.22)			
Case-related errors	–	–	–	–	–	–	1.89	2.41	1.09
							(1.90)	(2.08)	(1.23)

#### Orthographic competencies

The Hamburg Writing Test for first to ninth graders was used to measure orthographic competencies (German: Hamburger Schreibprobe 1–9; May, 2002). Age-appropriate versions for first, third, and eighth grade were used. The test requires the participants to write words and short sentences that are read aloud to them. The version for the eight graders also includes a text with mistakes to be corrected. The test has shown good re-test reliability (0.92–0.99) and high predictive validity (for example, correlations with school essay writing of *r*^2^ = 0.78–0.82; May, 2002). The test provides a profile for each student of the general spelling skills and specific spelling strategies, including alphabetical, orthographical, and morphological strategies. General spelling skills were measured by the number of correctly written words and graphemes. The test comprises 14 words/61 graphemes (Grade 1), 38 words/191 graphemes (Grade 3), and 49 words/339 graphemes (Grade 8) to compute general spelling ability. In the present data, higher scores reflected higher general spelling skills (i.e., more correct words and graphemes).

The alphabetical strategy refers to all word positions that can be spelled correctly by applying phonological rules. The number of positions in the different versions is 15 (Grade 1), 20 (Grade 3), and 30 (Grade 8). The orthographical strategy was coded for all word positions, for which knowledge of orthographic units, i.e., abstract letter-by-letter strings (Frith, 1985), is required. The orthographical strategy is distinguished from the alphabetical strategy as these word positions cannot be spelled correctly by applying alphabetical knowledge only (for example, a letter sound can be represented in several ways such as *x* can be written as *x, chs, ks, cks*, or *gs*). The number of positions in the different versions is 10 (Grade 1), 15 (Grade 3), and 41 (Grade 8). The morphological strategy is based on the number of correctly spelled critical morpheme positions. These are letter groups in a word that require morphological rather than phonological or orthographical knowledge, for example vowel mutations. Because it is the most advanced strategy, it was only coded for the students in Grade 8. The total number of critical morpheme positions in the test was 28. For analyses, the number of errors in applying each strategy were used as indicators for the specific writing and spelling strategies. Additionally, redundant elements (i.e., additional letters that indicate overgeneralization of alphabetic principles; for example, “ie” (Bield) instead of “i” (correct: Bild, in English: image, picture) were coded for the students in Grade 1 and Grade 3 and case-related errors (i.e., errors in capitalization of first letter in nouns and names) for the students in Grade 8, reflecting grade-appropriate expectations regarding the students' spelling. In the present data, higher scores indicated lower proficiency in applying specific spelling strategies, and more redundant elements and case-related errors in the students' writing samples.

### Analytic strategy

All analyses were conducted using the Bayesian approach with non-informative priors in Mplus 7.3 (Muthén and Muthén, 2014). The Bayes estimator compares an obtained value with a posterior probability distribution of predicted values (Kruschke, 2011). It is able to account for relatively small sample sizes and is robust to distributional assumptions of the estimated parameters of interest. Thus, it provides more trustworthy results than a traditional maximum likelihood estimator (Lee and Song, 2004; Muthén, 2010). In order to ensure model fit, we checked for convergence. Four chains were used in the Markov chain Monte Carlo estimation with a thinning option (20 draws) in order to control for autocorrelation. Good convergence is given, when the potential scale reduction factor keeps ranging around 1.00, while running the model with increasing numbers of iterations (Muthén, 2010). This was the case for all models analyzed and reported here. Meaningful estimates were indicated when the conditional confidence intervals of the fixed posterior distribution (Bayesian credibility intervals, BCI) for the estimates did not include zero. The BCIs can be interpreted similarly to those in traditional maximum likelihood estimation: A 90%-BCI refers to a significance level of *p* < 0.10, and a 95%-BCI to a significance level of *p* < 0.05.

The central goal of the present study was to test differences in the structural patterns of associations between two (i.e., between the boys and girls in Grade 3) or three subsamples (i.e., between the first, third, and eighth graders). Therefore, a multi-group approach with cross-group invariance testing was used. It allowed us to consider whether attention shifting contributed unique variance to different levels of spelling skills in each group separately. The advantage of cross-group invariance testing is that various structural parameters of interest (including means of predictors and outcomes, regression coefficients) in more than two subsamples can be tested against one another in one model. Compared to correlational analyses, this approach enables the investigation of relationships between multiple predictors and outcomes, accounts for possible co-variances between more than one indicator of interest, and considers several control variables that may potentially have a confounding effect on the relationship.

To examine cross-group invariance regarding age and sex in the prediction of orthographic competencies by the students' attention shifting (i.e., inverse efficiency score^2^), a three-step procedure was used for a correct estimation. Following the literature on measurement invariance analyses with Bayes (c.f., Van de Schoot et al., 2012; Muthén and Asparouhov, 2013), we have started with a full invariant model that sets all parameter equal across groups. Second, a full non-invariant model was tested that allowed variation in all parameter across groups. At the same time, difference tests for structural parameters of interest were included in order to identify meaningful differences between the groups. If a parameter was tested to be meaningfully different, then it was set free to vary between the groups in a third partial non-invariant model. The models were compared to each other in order to identify the best fitting model. For each set of analyses, the results of the best fitting model are reported in the Results section.

In more detail, the described procedure contained the following steps: In step 1, multiple regression models with configural equality constraints were estimated (i.e., full invariant models), holding all structural parameters of interest equal across the groups (i.e., regression coefficients, variable means). In step 2, non-invariant parameters were identified in models completely freeing the previously used equality constraints for the same parameters (i.e., full non-invariant models). For the analysis of cross-group invariance regarding age, difference tests between each group's structural parameter and its average across the three age groups (i.e., grade 1, 3, and 8) were used for identification. For the analysis of structural invariance regarding sex within age groups, non-invariant parameters were identified with difference tests between the two sex groups (i.e., girls and boys). In step 3, models holding all but the non-invariant parameters identified in step 2 equal across groups (i.e., partial invariant models) were conducted and compared to the full invariant models of step 1 and the respective full non-invariant models of step 2. For comparison, the Deviance Information Criterion (DIC; Gelman et al., 2004) was used. That is, the Bayesian model comparison criterion that is defined analogously to the Akaike Information Criterion (AIC) generated in ML estimation. The DIC takes the complexity of the model into account, i.e., estimated as the effective number of parameters. Models with the smallest DIC values were preferred (Muthén, 2010). Thereby, the Bayes approach detects cross-group non-invariance similar to Wald statistics with maximum-likelihood estimation (Muthén and Asparouhov, 2013).

All of the analyses controlled for child age, sex (0 = girls, 1 = boys) and home language (0 = child speaks German as home language, 1 = child speaks another language than German as primary home language). All variables were standardized (i.e., age, attention shifting, orthographic competence indicators) or dummy coded (i.e., sex, home language) before being entered into the analyses.

## Results

### Descriptive statistics

Descriptive statistics indicated that there was considerable variability in school-aged children's attention shifting and orthographic competencies, both within each grade and between grades (Table 1). The data showed that older students' attention shifting skills were higher than those of younger students (indicated by the smaller inverse efficiency scores among eighth graders compared to third graders and first graders). A similar pattern was found for students' word-level spelling and their proficiency to apply specific spelling strategies to their writing: Older students scored higher on the spelling test than younger students.

### The role of attention shifting for orthographic competencies: testing age invariance across grade levels

The first research question (i.e., Are there differences in the relation between attention shifting and orthographic competencies across the age groups?) tested for cross-group invariance of the relation between attention shifting and spelling across grade levels. The first model was specified as a full invariant model and resulted in a DIC of 1066.07. Next, a full non-invariant model was run to identify meaningful differences between structural parameters of interest. The full non-invariant model resulted in a DIC of 1045.07. The differences in several structural parameters between the grade levels are reported in Table 2.

**Table 2:** Results of difference tests for structural age invariance analyses in the full non-invariant model.

	**Grade 1 (*n* = 69)**			**Grade 3 (*****n*** = **121)**			**Grade 8 (*****n*** = **84)**		
	***B* (*SD*)**	**95% BCI**	**90% BCI**	***B* (*SD*)**	**95% BCI**	**90% BCI**	***B* (*SD*)**	**95% BCI**	**90% BCI**
**REGRESSION COEFFICIENTS**									
IE score on correct words	**0.13**	0.04,	0.06,	**−0.20**	−0.30,	−0.28,	0.07	−0.06,	−0.04,
	(0.05)	0.21	0.21	(0.05)	−0.11	−0.12	(0.06)	0.19	0.17
IE score on correct graphemes	**0.04**	0.01,	0.02,	**−0.05**	−0.07,	−0.07,	0.01	−0.03,	−0.03,
	(0.02)	0.07	0.07	(0.01)	−0.02	−0.03	(0.02)	0.04	0.03
IE score on alphabetical errors	0.01	−0.33,	−0.27,	0.18	−0.12,	−0.08,	−0.19	−0.59,	−0.56,
	(0.17)	0.33	0.29	(0.16)	0.49	0.44	(0.22)	0.25	0.15
IE score on orthographical errors	**−0.27**	−0.50,	−0.47,	**0.48**	0.21,	0.27,	−0.21	−0.52,	−0.47,
	(0.12)	−0.03	−0.08	(0.13)	0.72	0.69	(0.17)	0.13	0.08
**MEANS**									
Correct words	**−1.37**	−1.62,	−1.59,	**−0.18**	−0.33,	−0.31,	**1.55**	1.18,	1.25,
	(0.13)	−1.11	−1.17	(0.08)	−0.02	−0.05	(0.19)	1.93	1.88
Correct graphemes	**−1.34**	−1.40,	−1.39,	**−0.12**	−0.16,	−0.16,	**1.45**	1.36,	1.38,
	(0.04)	−1.26	−1.28	(0.02)	−0.07	−0.08	(0.05)	1.55	1.54
Alphabetical errors	0.36	−0.51,	−0.32,	**0.52**	0.01,	0.09,	−0.88	−2.14,	−1.93,
	(0.42)	1.15	1.08	(0.26)	1.04	0.94	(0.61)	0.32	0.10
Orthographical errors	**1.13**	0.47,	0.59,	**0.40**	0.01,	0.06,	**−1.52**	−2.61,	−2.35,
	(0.34)	1.83	1.73	(0.21)	0.80	0.74	(0.51)	−0.54	−0.66
IE score	**1.21**	1.04,	1.08,	**−0.14**	−0.26,	−0.24,	**−1.06**	−1.20,	−1.17,
	(0.08)	1.36	1.35	(0.06)	−0.03	−0.05	(0.07)	−0.94	−0.96

*IE, Inverse Efficiency. Structural invariance between age groups was tested using Bayes estimation. Analyses controlled for child age, sex, and migration background. Bold numbers indicate meaningful unstandardized coefficients (posterior standard deviation) with a Bayesian Credibility Interval (BCI) excluding zero. The coefficients display the difference estimates between the respective structural parameter and its average across the three age groups (i.e., difference value = group parameter – parameter average)*.

**Figure 1:**
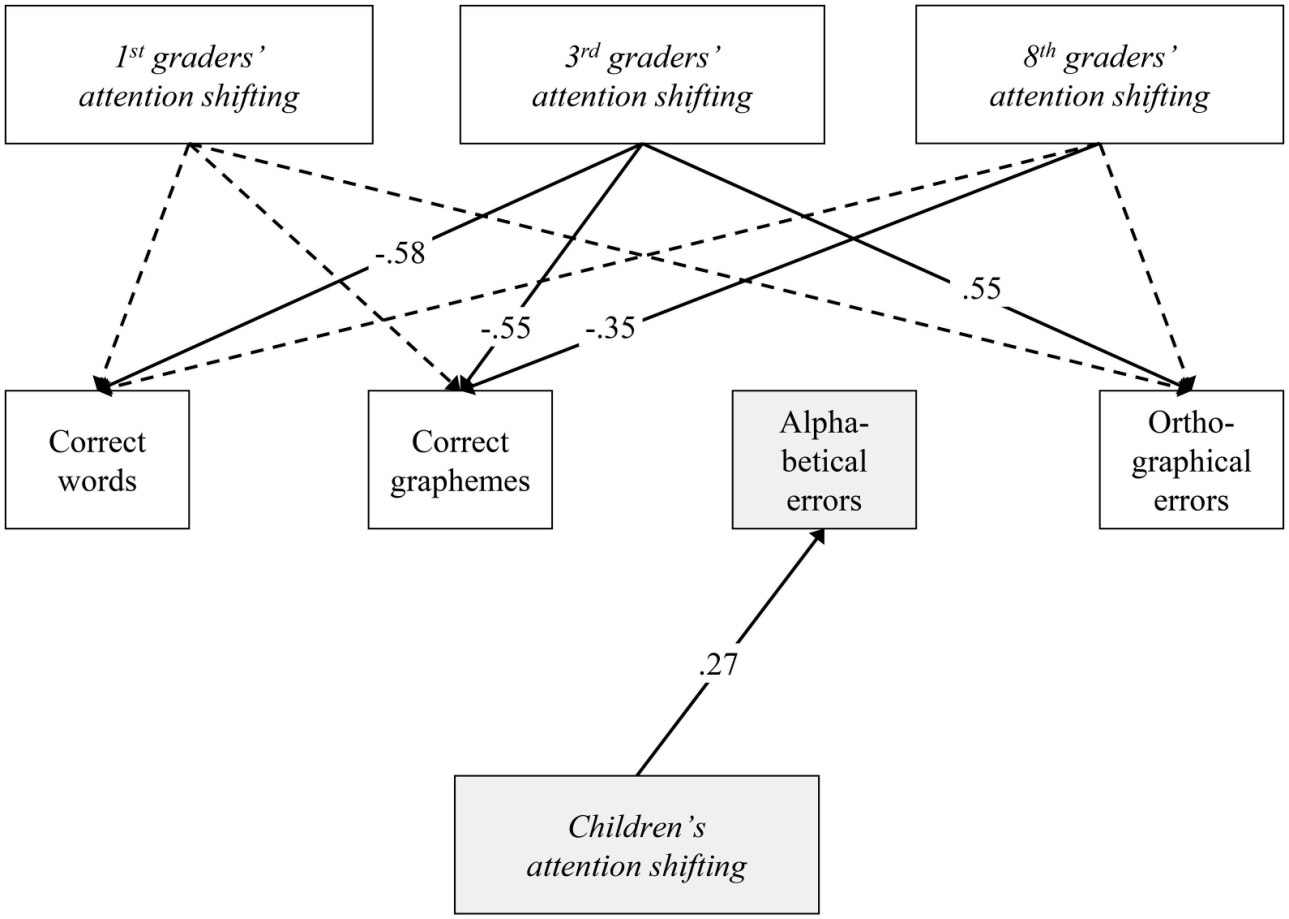
Favored structural partial invariant model regarding age for prediction of orthographic competences. Structural age invariance was tested using Bayes estimation. Analyses control for child age, gender, and migration background. Solid lines indicate meaningful relations (standardized coefficients), each with a Bayesian Credibility Interval excluding zero (see Tables 2, 3). Gray boxes highlight age invariant relations.

To further investigate these differences, a partial invariant model was run. The model released all parameters that were found to be different in the previous model but set all remaining parameters to be equal across grade levels. The DIC of the partial invariant model was lowest (DIC = 1043.88). Therefore, this model was preferred over the full non-invariant model. The partial invariant model revealed a meaningful relation across all grade levels (i.e., invariant regression coefficient) between the students' attention shifting and their proficiency in using the alphabetical strategy in their writing (Figure 1, bottom). For all of the students, better attention shifting (indicated by a lower inverse efficiency score) was related to fewer errors in alphabetical spelling. Furthermore, differences across grade levels were identified (as indicated by variant regression coefficients; Table 3 and Figure 1). Meaningful relations emerged between the third-grade students' attention shifting and word-level spelling skills, and between attention shifting and the use of the orthographic spelling strategy. Higher levels of the third graders' attention shifting skills were associated with more correctly written words and graphemes, and fewer orthographic errors. For the eighth graders, attention shifting was related to general spelling skills, with higher attention shifting skills being associated with more correctly written graphemes. The detailed model parameters (regression coefficients, posterior standard deviation, Bayesian credibility intervals) are reported in Table 3.

**Table 3:** Favored structural partial invariant model regarding age for prediction of orthographic competences.

	**Correct words**				**Correct graphemes**				**Alphabetical errors**				**Orthographical errors**			
	***B* (*SD)***	**95%BCI**	**90%BCI**	**β (*SD*)**	***B* (*SD*)**	**95%BCI**	**90%BCI**	**β (*SD*)**	***B* (*SD*)**	**95%BCI**	**90%BCI**	**β (*SD*)**	***B* (*SD*)**	**95%BCI**	**90%BCI**	**β (*SD*)**
**ATTENTION SHIFTING**																
Grade 1	−0.02	−0.09,	−0.08,	−0.05	0.01	−0.04,	−0.03,	0.02	**0.41** (0.11)	0.17,	0.23,	0.27	0.09	−0.13,	−0.10,	0.07
(*n* = 69)	(0.04)	0.05	0.03	(0.08)	(0.02)	0.03	0.03	(0.15)		0.61	0.60	(0.07)	(0.11)	0.30	0.28	(0.09)
	Cohen's *f^2^* = 0.03				Cohen's *f^2^* = 0.05				Cohen's *f^2^* = 0.09				Cohen's *f^2^* = 0.03			
Grade 3	**−0.32**	−0.42,	−0.40,	−0.58	**−0.08**	−0.10,	−0.09,	−0.55	**0.41**	0.17,	0.23,	0.27	**0.79**	0.55,	0.58,	0.55
(*n* = 121)	(0.05)	−0.24	−0.24	(0.07)	(0.01)	−0.05	−0.06	(0.07)	(0.11)	0.61	0.60	(0.07)	(0.13)	1.06	1.00	(0.07)
	Cohen's *f^2^* = 0.55				Cohen's *f^2^* = 0.48				Cohen's *f^2^* = 0.09				Cohen's *f^2^* = 0.48			
Grade 8	−0.12	−0.27,	−0.24,	−0.26	**−0.04**	−0.08,	−0.07,	−0.35	**0.41** (0.11)	0.17,	0.23,	0.27	0.27	−0.10,	−0.06,	0.22
(*n* = 84)	(0.08)	0.03	0.01	(0.15)	(0.02)	−0.01	−0.01	(0.14)		0.61	0.60	(0.07)	(0.21)	0.73	0.63	(0.16)
	Cohen's *f^2^ = 0.10*				Cohen's *f^2^ = 0.18*				Cohen's *f^2^ = 0.09*				Cohen's *f^2^ = 0.08*			

*Structural invariance between age groups was tested using Bayes estimation. Analyses controlled for child age, sex, and migration background. Bold numbers indicate meaningful unstandardized coefficients (posterior standard deviation) with a Bayesian Credibility Interval (BCI) excluding zero. Cohen's f^2^ represents the effect size for attention shifting on the respective outcome, with f^2^ = 0.02 rated as small, f^2^ = 0.15 as medium, and f^2^ = 0.35 as strong effect*.

### The role of attention shifting for orthographic competencies: testing sex invariance within each grade level

**Figure 2:**
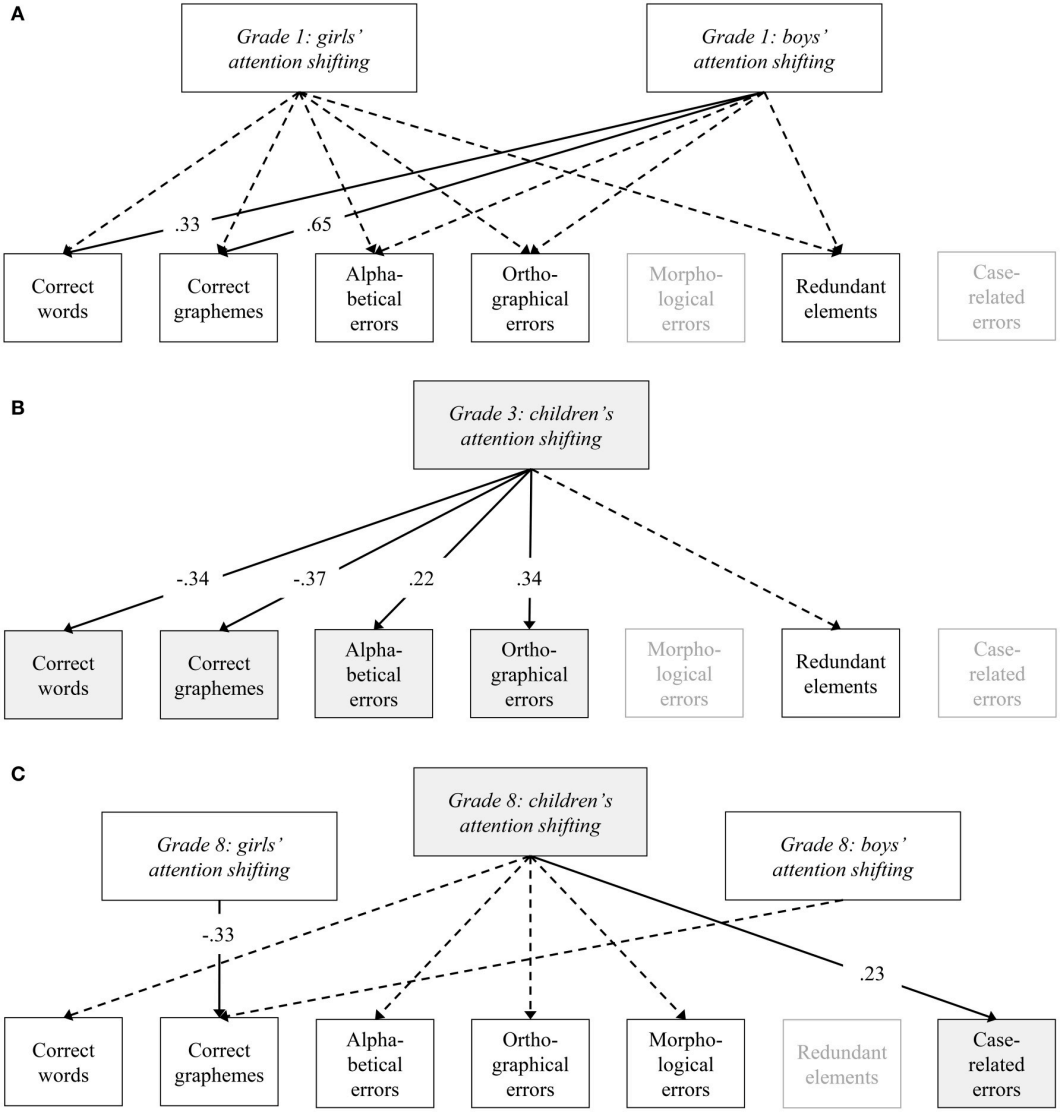
**(A)** Favored structural full non-invariant model regarding gender for prediction of orthographic competences in grade 1. **(B)** Favored structural full invariant model regarding gender for prediction of orthographic competences in grade 3. **(C)** Favored structural partial invariant model regarding gender for prediction of orthographic competences in grade 8. **(A–C)** Structural gender invariance was tested using Bayes estimation. Analyses control for child age, gender, and migration background. Solid lines indicate meaningful relations (standardized coefficients), each with a Bayesian Credibility Interval excluding zero (see Tables 1–4). Gray boxes highlight gender invariant relations.

To answer the second research question (i.e., Does attention shifting relate to orthographic competencies equally for boys and girls within each age group?), separate sets of models were run to test structural sex invariance within Grade 1, Grade 3, and Grade 8. For the first-grade students, the full invariant model across sex resulted in a DIC of 1164.21. The corresponding full non-invariant model revealed a DIC of 1137.34, showing a meaningful difference for the regression coefficient between attention shifting and general spelling skills for boys and girls (i.e., number of correct graphemes; Table 4). The partial invariant model releasing the corresponding parameter showed a DIC of 1138.13. Thus, the full non-invariant model was preferred. The model indicated a meaningful relation between attention shifting and general spelling skills indicating that higher attention shifting was associated with fewer correctly written words and graphemes (Table 5 and Figure 2A). However, the relation was only found for the boys in Grade 1 but not for the girls.

**Table 4:** Results of difference test for structural sex invariance analyses of the full non-invariant models in grade 1, 3, and 8.

	**Grade 1 (*****n*** = **69)**			**Grade 3 (*****n*** = **121)**			**Grade 8 (*****n*** = **84)**		
	***B* (*SD*)**	**95% BCI**	**90% BCI**	***B* (*SD*)**	**95% BCI**	**90% BCI**	***B* (*SD*)**	**95% BCI**	**90% BCI**
**REGRESSION COEFFICIENTS**									
IE score on correct words	−0.19	−0.79,	−0.61,	−0.29	−0.75,	−0.60,	−0.31	−0.85,	−0.69,
	(0.26)	0.17	0.17	(0.20)	0.09	0.05	(0.27)	0.16	0.14
IE score on correct graphemes	**−0.37**	−0.85,	−0.69,	−0.26	−0.65,	−0.58,	**−0.39**	−0.83,	−0.83,
	(0.20)	−0.05	−0.05	(0.20)	0.14	0.09	(0.25)	0.10	−0.05
IE score on alphabetical errors	−0.15	−0.65,	−0.55,	0.08	−0.30,	−0.24,	0.19	−0.38,	−0.38,
	(0.28)	0.41	0.35	(0.20)	0.49	0.42	(0.30)	0.69	0.58
IE score on orthographical errors	−0.26	−0.68,	−0.61,	0.22	−0.20,	−0.12,	0.27	−0.36,	−0.18,
	(0.24)	0.19	0.15	(0.20)	0.61	0.56	(0.30)	0.79	0.79
IE score on morphological errors	–	–	–	–	–	–	0.25	−0.11,	−0.11,
							(0.21)	0.66	0.58
IE score on redundant elements	−0.21	−0.50,	−0.49,	0.39	−0.41,	−0.35,	–	–	–
	(0.18)	0.20	0.09	(0.42)	1.23	1.04			
IE score on case-related errors	–	–	–	–	–	–	−0.10	−1.03,	−0.73,
							(0.51)	0.98	0.93
**MEANS**									
Correct words	−0.29	−0.88,	−0.73,	−0.05	−0.44,	−0.36,	**−0.47**	−0.90,	−0.81,
	(0.27)	0.12	0.12	(0.19)	0.31	0.27	(0.23)	−0.04	−0.08
Correct graphemes	−0.09	−0.60,	−0.51,	−0.15	−0.52,	−0.45,	**−0.46**	−0.88,	−0.88,
	(0.24)	0.33	0.26	(0.19)	0.24	0.18	(0.23)	0.05	−0.12
Alphabetical errors	0.12	−0.52,	−0.43,	−0.25	−0.64,	−0.56,	0.28	−0.22,	−0.09,
	(0.30)	0.71	0.51	(0.20)	0.15	0.10	(0.25)	0.71	0.71
Orthographical errors	0.12	−0.44,	−0.30,	0.08	−0.26,	−0.24,	0.06	−0.37,	−0.33,
	(0.29)	0.68	0.63	(0.20)	0.51	0.39	(0.23)	0.47	0.39
Morphological errors	–	–	–	–	–	–	−0.01	−0.41,	−0.38,
							(0.22)	0.41	0.28
Redundant elements	−0.25	−0.66,	−0.52,	−0.09	−0.85,	−0.70,	–	–	–
	(0.19)	0.10	0.10	(0.40)	0.73	0.62			
Case-related errors	–	–	–	–	–	–	**1.40**	0.42,	0.60,
							(0.47)	2.29	2.13
IE score	0.20	−0.73,	−0.55,	0.30	−0.08,	−0.03,	0.01	−0.51,	−0.43,
	(0.50)	1.29	1.04	(0.20)	0.70	0.63	(0.25)	0.42	0.37

*IE, Inverse Efficiency. Structural invariance between sex groups was tested using Bayes estimation. Analyses controlled for child age and migration background. Bold numbers indicate meaningful unstandardized coefficients (posterior standard deviation) with a Bayesian Credibility Interval (BCI) excluding zero. The coefficients display the difference estimates between girls' and boys' respective parameters (i.e., difference value = girls' parameter – boys' parameter)*.

**Table 5:** Favored structural full non-invariant model regarding sex for prediction of orthographic competences in grade 1.

	**Correct words**				**Correct graphemes**				**Alphabetical errors**				**Orthographical errors**				**Redundant elements**			
	***B* (*SD*)**	**95% BCI**	**90% BCI**	**β (*SD*)**	***B* (*SD*)**	**95% BCI**	**90% BCI**	**β (*SD*)**	***B* (*SD*)**	**95% BCI**	**90% BCI**	**β (*SD*)**	***B* (*SD*)**	**95% BCI**	**90% BCI**	**β (*SD*)**	***B* (*SD*)**	**95% BCI**	**90% BCI**	**β (*SD*)**
**ATTENTION SHIFTING IN GRADE 1**																				
Girls	0.04	−0.50,	−0.37,	0.06	0.11	−0.27,	−0.24,	−0.18	−0.13	−0.60,	−0.52,	−0.18	−0.07	−0.47,	−0.42,	−0.10	−0.19	−0.44,	−0.41,	−0.42
(*n* = 29)	(0.23)	0.40	0.39	(0.32)	(0.18)	0.43	0.35	(0.3)	(0.27)	0.46	0.35	(0.35)	(0.22)	0.39	0.29	(0.29)	(0.14)	0.14	0.07	(0.28)
	Cohen's *f^2^* = 0.30				Cohen's *f^2^* = 0.26				Cohen's *f^2^* = 0.22				Cohen's *f^2^* = 0.15				Cohen's *f^2^* = 0.25			
Boys	**0.22**	0.01,	0.04,	0.33	**0.46**	0.27,	0.31,	0.65	0.06	−0.19,	−0.15,	0.08	0.19	−0.04,	−0.01,	0.29	0.02	−0.14,	−0.10,	0.05
(*n* = 40)	(0.11)	0.43	0.41	(0.14)	(0.11)	0.68	0.65	(0.11)	(0.13)	0.30	0.25	(0.17)	(0.12)	0.44	0.39	(0.16)	(0.08)	0.17	0.15	(0.17)
	Cohen's *f^2^* = 0.41				Cohen's *f^2^* = 1.17				Cohen's *f^2^* = 0.10				Cohen's *f^2^* = 0.14				Cohen's *f^2^* = 0.06			

*Structural sex invariance within age groups was tested using Bayes estimation. Analyses controlled for child age and migration background. Bold numbers indicate meaningful unstandardized coefficients (posterior standard deviation) with a Bayesian Credibility Interval (BCI) excluding zero. Cohen's f^2^ represents the effect size for attention shifting on the respective outcome, with f^2^ = 0.02 rated as small, f^2^ = 0.15 as medium, and f^2^ = 0.35 as strong effect*.

For the third-grade group, compared to the full non-invariant model (DIC = 2087.60) the full invariant model across sex showed a lower DIC (DIC = 2079.98). No meaningful differences were found in the difference tests of the full non-invariant model and, thus, the full invariant model was preferred (Table 4). Among third grade students of both sexes, attention shifting showed meaningful relations with general spelling skills (i.e., number of correctly written words and graphemes) and with students' proficiency in the use of specific spelling strategies (Table 6 and Figure 2B). Higher levels of attention shifting were related with higher general spelling skills and with less alphabetical and orthographic errors in students' writing sample.

**Table 6:** Favored structural full invariant model regarding sex for prediction of orthographic competences in grade 3.

	**Correct words**				**Correct graphemes**				**Alphabetical errors**				**Orthographical errors**				**Redundant elements**			
	***B* (*SD*)**	**95% BCI**	**90% BCI**	**β (*SD*)**	***B* (*SD*)**	**95% BCI**	**90% BCI**	**β (*SD*)**	***B* (*SD*)**	**95% BCI**	**90% BCI**	**β (*SD*)**	***B* (*SD*)**	**95% BCI**	**90% BCI**	**β (*SD*)**	***B* (*SD*)**	**95% BCI**	**90% BCI**	**β (*SD*)**
**ATTENTION SHIFTING IN GRADE 3**																				
Girls	**−0.35**	−0.55,	−0.52,	−0.34	**−0.38**	−0.57,	−0.55,	−0.37	**0.23**	0.01,	0.06,	0.22	**0.35**	0.15,	0.18,	0.34	−0.08	−0.47,	−0.39,	−0.04
(*n* = 59)	(0.10)	−0.16	−0.20	(0.10)	(0.10)	−0.18	−0.23	(0.09)	(0.10)	0.41	0.40	(0.10)	(0.10)	0.53	0.51	(0.09)	(0.22)	0.38	0.31	(0.10)
	Cohen's *f^2^* = 0.17				Cohen's *f^2^* = 0.20				Cohen's *f^2^* = 0.09				Cohen's *f^2^* = 0.18				Cohen's *f^2^* = 0.05			
Boys	**−0.35**	−0.55,	−0.52,	−0.34	**−0.38**	−0.57,	−0.55,	−0.37	**0.23**	0.01,	0.06,	0.22	**0.35**	0.15,	0.18,	0.34	−0.08	−0.47,	−0.39,	−0.04
(*n* = 62)	(0.10)	−0.16	−0.20	(0.10)	(0.10)	−0.18	−0.23	(0.09)	(0.10)	0.41	0.40	(0.10)	(0.10)	0.53	0.51	(0.09)	(0.22)	0.38	0.31	(0.10)
	Cohen's *f^2^* = 0.17				Cohen's *f^2^* = 0.20				Cohen's *f2* = 0.09				Cohen's *f^2^* = 0.18				Cohen's *f^2^* = 0.05			

*Structural sex invariance within age groups was tested using Bayes estimation. Analyses control for child age and migration background. Bold numbers indicate meaningful unstandardized coefficients (posterior standard deviation) with a Bayesian Credibility Interval (BCI) excluding zero. Cohen's f^2^ represents the effect size for attention shifting on the respective outcome, with f^2^ = 0.02 rated as small, f^2^ = 0.15 as medium, and f^2^ = 0.35 as strong effect*.

With regard to the eighth-grade students, the full invariant model across sex yielded a DIC of 1931.79. The full non-invariant model showed a DIC of 1943.89. The full non-invariant model, however, indicated that the boys compared to the girls had higher word-level spelling skills (i.e., produced more correct words and graphemes in their writing sample) and made less case-related errors. In addition, the regression coefficients of attention shifting on the number of correct graphemes differed for the boys and the girls. Therefore, a partial invariant model was run that released the respective parameters. The model showed the lowest DIC (1928.56) and was therefore considered the preferred model. In the partial invariance model, the girls' but not the boys' attention shifting was meaningfully related with the number of correct graphemes. Thus, among the eighth-grade students, higher attention shifting skills were related with higher general spelling skills at the level of the grapheme, but only for the girls. At the same time, a meaningful relation between attention shifting and case-related errors was found for both, the boys and girls (see Table 7 and Figure 2C). Higher attention shifting was related with higher proficiency in capitalization for both sexes.

**Table 7:** Favored structural partial invariant model regarding sex for prediction of orthographic competences in grade 8.

	**Correct words**				**Correct graphemes**				**Alphabetical errors**				**Orthographical errors**				**Morphological errors**				**Case-related errors**			
	***B* (*SD*)**	**95% BCI**	**90% BCI**	**β (*SD*)**	***B* (*SD*)**	**95% BCI**	**90% BCI**	**β (*SD*)**	***B* (*SD*)**	**95% BCI**	**90% BCI**	**β (*SD*)**	***B* (*SD*)**	**95% BCI**	**90% BCI**	**β (*SD*)**	***B* (*SD*)**	**95% BCI**	**90% BCI**	**β (*SD*)**	***B* (*SD*)**	**95% BCI**	**90% BCI**	**β (*SD*)**
**ATTENTION SHIFTING IN GRADE 8**																								
Girls	−0.16	−0.38,	−0.36,	−0.15	**−0.35**	−0.61,	−0.57,	−0.33	0.12	−0.12,	−0.08,	0.11	0.14	−0.09,	−0.07,	0.13	0.12	−0.09,	−0.05,	0.14	**0.45**	0.02,	0.08,	0.23
(*n* = 51)	(0.12)	0.09	0.02	(0.11)	(0.14)	−0.07	−0.10	(0.12)	(0.12)	0.35	0.32	(0.11)	(0.12)	0.39	0.33	(0.11)	(0.10)	0.29	0.27	(0.11)	(0.22)	0.86	0.78	(0.11)
	Cohen's *f^2^* = 0.06				Cohen's *f^2^* = 0.15				Cohen's *f^2^* = 0.05				Cohen's *f^2^* = 0.06				Cohen's *f^2^* = 0.05				Cohen's *f^2^* = 0.09			
Boys	−0.16	−0.38,	−0.36,	−0.15	−0.08	−0.41,	−0.33,	−0.07	0.12	−0.12,	−0.08,	0.11	0.14	−0.09,	−0.07,	0.13	0.12	−0.09,	−0.05,	0.14	**0.45**	0.02,	0.08,	0.23
(*n* = 33)	(0.12)	0.09	0.02	(0.11)	(0.18)	0.27	0.25	(0.17)	(0.12)	0.35	0.32	(0.11)	(0.12)	0.39	0.33	(0.11)	(0.10)	0.29	0.27	(0.11)	(0.22)	0.86	0.78	(0.11)
	Cohen's *f^2^* = 0.06				Cohen's *f^2^* = 0.05				Cohen's *f^2^* = 0.05				Cohen's *f^2^* = 0.06				Cohen's *f^2^* = 0.05				Cohen's *f^2^* = 0.09			

*Structural sex invariance within age groups was tested using Bayes estimation. Analyses control for child age and migration background. Bold numbers indicate meaningful unstandardized coefficients (posterior standard deviation) with a Bayesian Credibility Interval (BCI) excluding zero. Cohen's f^2^ represents the effect size for attention shifting on the respective outcome, with f^2^ = 0.02 rated as small, f^2^ = 0.15 as medium, and f^2^ = 0.35 as strong effect*.

## Discussion

The present study examined associations between attention shifting, word-level spelling skills and specific spelling strategies in a group of first, third, and eighth grade students. In general, attention shifting was related to spelling outcomes for all of the students. The associations were particularly strong among the third-grade students. In this age group, there were no sex differences in the relations between attention shifting and spelling outcomes. Among the first- and eighth-grade students, however, findings suggest sex differences in the relationship between attention shifting and general, i.e., word-level spelling. While for the eighth-grade girls, higher attention shifting skills were related to higher general spelling skills, the opposite was true for the first-grade boys, i.e., higher attention shifting skills were related to lower general spelling skills. Together, the findings add to the literature by suggesting that the pattern of associations between attention shifting and various orthographic competencies differs across age groups and by sex.

### Age-related similarities and differences in the pattern of associations

The current study expanded on previous research by providing initial evidence of age-related similarities and differences in the pattern of associations between attention shifting, one core component of executive functions, and spelling, that depended on whether general (i.e., word-level) spelling skills or specific spelling strategies were examined. One obvious hypothesis is that shifting abilities should be equally important for word-level spelling across different stages of spelling development. This is because word-level spelling requires shifting between several mental tasks, including “listening to the dictation, writing words either by retrieving their orthographic form from memory or by applying phoneme-grapheme correspondence rules […], and verifying their production” (Lubin et al., 2016, p. 453) that should not differ between beginning and proficient spellers. In our study, attention shifting was related to general spelling among the third-grade and the eighth-grade students (though the associations among the eighth graders were only at the level of the grapheme). However, we did not find that attention shifting was related to general spelling skills among the first-grade students in comparison with the other age groups.

It could be that our findings may reflect specifics of spelling instruction in schools. In Germany, instructional emphasis in the early elementary grades is on phonemic spelling with teachers predominantly using words that have consistent one-to-one grapheme-phoneme-correspondence (Valtin, 1997). This results in a high probability of correct spelling. Thus, first graders' spelling might not draw heavily on attention shifting. The relative contribution of attention shifting to spelling, however, might change when students enter Grade 3 and are expected to apply spelling rules in order to master the spelling of unfamiliar words that contain inconsistencies between sound and orthographic patterns (Valtin, 1997; Moll et al., 2009). That is the time when individual differences in spelling become more prominent as students wrongly apply specific orthographic regularities where they are not needed (Valtin, 1997; Moll et al., 2009). Our findings suggest that attention shifting skills might provide a potential explanation for individual differences in spelling among older students. This assumption is supported by previous work reporting that shifting abilities but not working memory and inhibition accounted for variance in fourth graders' spelling skills (Lubin et al., 2016). Although, our study is among the first to investigate the relation between attention shifting and spelling outcomes at different stages of spelling acquisition, longitudinal research following children from middle childhood into adolescence is needed to better understand the (possibly changing) role that attention shifting plays in word-level spelling.

With regard to specific spelling strategies, attention shifting was related to the alphabetical strategy for all of the students across grades. That is, independent of the students' developmental level of spelling proficiency, faster but accurate performance on the DCCS was associated with less errors in applying the alphabetic principle to one's writing. A possible explanation could be that shifting abilities influence how spelling-relevant information is processed (Buchholz and Davies, 2005). For both beginning and proficient spellers, the ability to understand and apply the alphabetic principle has been linked to phonological processing (Dich and Cohn, 2013; Moll et al., 2014; Yeong et al., 2014). Deficits in attention were associated with impairments in phonological processing skills (Facoetti et al., 2010). Across different stages of spelling acquisition, shifting abilities may be important for the alphabetic spelling strategy because of the relationship with phonological processing skills. Future research should thus include this construct when studying associations between attention shifting and spelling skills.

An unexpected finding was that attention shifting was related to the orthographic strategy only among the third grade students. In the German orthography, most spelling errors are caused by orthographic deficits (Moll et al., 2009). That is, “phoneme-grapheme conversion results in phonologically adequate but orthographically incorrect spellings” (Moll et al., 2009, p. 4). Consequently, attention shifting should be equally important for orthographic processing at all stages of spelling acquisition due to its importance for one's ability to differentiate between various representations of letter-sound correspondences. However, our findings could point to age-related changes in the association between attention shifting and orthographic spelling. Shifting abilities undergo rapid developmental changes from middle childhood to early adolescence (Anderson, 2010). During the same period, children learn to apply orthographic knowledge to their spelling (e.g., Cunningham et al., 2002; Sprenger-Charolles et al., 2003; Roman et al., 2009; Yeong et al., 2014). Shifting abilities may be particularly relevant for orthographic skills during the initial phase of building up proficiency in orthographic processing and less relevant later in development. As discussed above, from the beginning of Grade 3 students are increasingly exposed to inconsistencies of the German spelling system while they still lack adequate orthographic knowledge to cope with these inconsistencies (Moll et al., 2009). One possible explanation for our finding is that at this stage of spelling acquisition students with low attention shifting may have difficulties in building up orthographic proficiency. However, the directionality of the examined associations could not fully be identified due to the cross-sectional nature of the data. Alternatively, attention shifting may be indirectly related to orthographic skills through word-specific knowledge. Moll and colleagues (Moll et al., 2009, 2014) argue that the capacity of one's orthographic lexicon is an important predictor of orthographically correct spellings. Attention shifting may be relevant for more specific mechanisms underlying orthographic spelling. For example, correct spelling requires recall activity that is related to shifting abilities because students need to switch between different levels of analyzing words (Aram et al., 2014; Lubin et al., 2016). Further research is needed to investigate the mechanisms that may explain age-related differences in the relation between attention shifting and specific spelling strategies.

### Sex differences in the associations between attention shifting and spelling outcomes

We found that the relation between attention shifting and general (i.e., word-level) spelling skills differed for boys and girls but only among first and eighth graders. In contrast, no sex differences were found in the association between attention shifting and specific spelling strategies. Among first-grade boys only, slower and less accurate performance on the DCCS was associated with higher spelling skills at the word level (i.e., more correctly written words and graphemes). Our data does not explain why this was the case. Children with lower shifting abilities may benefit from the emphasis on words with consistent one-to-one grapheme-phoneme-correspondence which might not draw heavily on attention shifting. The emphasis on one-to-one grapheme-phoneme-correspondence is typical of spelling instruction in the early elementary years in Germany (Valtin, 1997). The boys with lower shifting abilities in our first-grade group could thus still be able to produce correct spellings because errors in the words' phoneme-grapheme conversion are less likely. In the eighth-grade sample, higher attention shifting skills were related to the girls' (but not boys') higher general spelling skills. These findings speak to the well documented achievement advantage for girls in secondary school (Bos et al., 2007; Quenzel and Hurrelmann, 2010).

The present findings could reflect sex differences in the strategies that boys and girls apply when directing their attention (Sobeh and Spijkers, 2012, 2013). There is initial evidence that boys perform faster in attention shifting tasks whereas girls demonstrate better accuracy (Sobeh and Spijkers, 2012, 2013). Influenced by biological factors, “accuracy of performance seems to develop earlier than the speed of performance” (Sobeh and Spijkers, 2013, p. 332). Sex differences in developmental trajectories of attention strategies may give girls an advantage to apply their shifting skills in a way that benefits their spelling, whereas for boys this might not be the case. It may even adversely affect their spelling, in particular, during the early elementary years. However, more research is needed to disentangle possible age-related changes in the mechanisms underlying sex differences in attention shifting and its relations to achievement outcomes, such as spelling.

An alternative explanation for the detected sex differences in the associations could be a measurement artifact. In the present study, spelling was measured using a conventional paper-and-pencil spelling test. Results of a study that compared spelling performance on paper-and-pencil tests and computerized tests suggest that boys perform better on computerized tests (Horne, 2007). Horne (2007) argued that using a computer enhances boys' motivation to engage with the test which results in more accurate performance. In addition, the sex differences could be due to differences in children's hand writing abilities which could not be tested in this study. Thus, our spelling measure may have underestimated boys' spelling level which might have resulted in the negative relation between attention shifting and spelling for the first graders.

### Practical implications

The present results have several implications for educational practice. First, students' spelling proficiency may be improved by enhancing teachers' awareness of the importance of attention shifting for spelling skills. Several studies have shown that teachers' pedagogical knowledge influences classroom practices and the quality of instruction which in turn has an effect on students' learning and performance (e.g., Metzler and Woessmann, 2012; Kunter et al., 2013; König and Pflanzl, 2016). Second, efforts to improve students' spelling might benefit from a focus on attention shifting. Intervention studies of school-based programs reported improvements in students' executive functions with particular strong effects on children with executive function difficulties (e.g., Diamond et al., 2007; Flook et al., 2010; Diamond and Lee, 2011). Positive effects of reading-specific flexibility exercises (focusing on shifting attention between phonological and semantic dimensions) that were completed with students as part of regular classroom activities have been shown to improve elementary students' reading (Cartwright, 2006). Such programs might be particularly relevant for third grade students who have to master the transition from phonological to orthographic spelling. Thus, providing a learning environment with ample opportunities to learn and practice executive function skills may facilitate students' spelling acquisition. Finally, many educational tests use general (i.e., word-level) spelling scores to classify students into good and poor spellers. As a consequence, spelling instruction has centered around the spelling of words. However, students might benefit from a focus on various specific spelling strategies after they have acquired foundational knowledge of letter-sound correspondences (Keunig and Verhoeven, 2008).

### Limitations and future directions

Although, our study addresses several limitations of prior work by including a wide age range and using age-appropriate versions of the same tasks to measure attention shifting and spelling outcomes, some caveats should be noted when interpreting the results. Our results suggested age-related differences in the associations between attention shifting and spelling outcomes. Unobserved variables such as intelligence and socioeconomic status may have accounted for the associations between attention shifting and spelling outcomes. Limitations in the available data did not allow us to control for these variables in the present analyses. However, previous research suggests that executive functions predict academic outcomes above and beyond intelligence (Duckworth and Seligman, 2005; Lubin et al., 2016) and socioeconomic status (Moffitt et al., 2011).

A second limitation is that the cross-sectional design of our study did not allow us to follow individual students over time. Thus, the differences between age groups may be due to student characteristics specific to each age group that could not be controlled in the present analyses. Future research should use a longitudinal design to investigate developmental trajectories of the relation between attention shifting and spelling which could also give insights into possible bidirectional associations of these two developing skills from childhood to adolescence. There is emerging evidence of simultaneous growth and reciprocal relations between executive functions and literacy skills during the early childhood years (Bohlmann et al., 2015; Slot and von Suchodoletz, submitted), but research from middle childhood into adolescence is still scarce.

Additional limitations concern the small sample sizes for each age group, in particular, when analyzing sex differences within each sample. Further studies are needed to confirm the findings with a larger sample. Another limiting fact refers to the missing analogy in covered characteristics between the measures used to assess spelling and attention shifting. While the latter was assessed with a process-related measure (inverse efficiency score produced by percentage of correct trials and their reaction times of the DCCS), we had no information on, for example, writing speed and error handling during the process of writing. A better congruence between measures should be a focus in future research. Finally, to get a more accurate picture of the relative contribution of attention shifting to academic outcomes, it would be beneficial to include other core executive functions (e.g., working memory and inhibition) as well as further outcome variables (e.g., reading skills).

## Conclusion

Together with previous research, the present cross-sectional findings emphasize the important role of attention shifting, one core component of executive functions, for German students' spelling skills in middle childhood and early adolescence. Efforts aimed at improving shifting abilities may help students to reach grade-level spelling proficiency. The findings are relevant for teacher education and professional development as they emphasize the necessity to enable teachers to tailor instructions to both reinforcing students' academic skills and their executive functions in order to improve school achievement. Finally, the study goes beyond previous research by providing an age- and sex-specific approach to the relation between attention shifting and spelling. Similarities and differences in the pattern of associations were identified that depend on students' age, sex, and specific spelling skill measured, thus, identifying possible developmental processes that should be examined by future research.

## Author contributions

AvS conceptualized and designed the study. The data collection was conducted by AvS and IS. AF performed the statistical analyses. AvS and AF composed the paper. IS contributed to the writing of the manuscript.

### Conflict of interest statement

The authors declare that the research was conducted in the absence of any commercial or financial relationships that could be construed as a potential conflict of interest.

## Footnotes

^1^Program for International Student Assessment (PISA).

^2^All models were also estimated using reaction time as a measure of attention shifting. Results were found to be similar when using reaction time data.
